# A framework for improving wheat spike development and yield based on the master regulatory TOR and SnRK gene systems

**DOI:** 10.1093/jxb/erac469

**Published:** 2022-12-07

**Authors:** Richard B Flavell

**Affiliations:** International Wheat Yield Partnership, 1500 Research Parkway, College Station, TX 77843, USA; University of Nottingham, UK

**Keywords:** Breeding, grain yield, kinases, spike development, stress, wheat

## Abstract

The low rates of yield gain in wheat breeding programs create an ominous situation for the world. Amongst the reasons for this low rate are issues manifested in spike development that result in too few spikelets, fertile florets, and therefore grains being produced. Phases in spike development are particularly sensitive to stresses of various kinds and origins, and these are partly responsible for the deficiencies in grain production and slow rates of gain in yield. The diversity of developmental processes, stresses, and the large numbers of genes involved make it particularly difficult to prioritize approaches in breeding programs without an overarching, mechanistic framework. Such a framework, introduced here, is provided around the master regulator target of rapamycin and sucrose non-fermenting-1 (SNF1)-related protein kinase complexes and their control by trehalose-6-phosphate and other molecules. Being master regulators of the balance between growth and growth inhibition under stress, these provide genetic targets for creating breakthroughs in yield enhancement. Examples of potential targets and experimental approaches are described.

## Teaser

The roles of protein phosphorylation and dephosphorylation during wheat spike development are reviewed for the purpose of defining suitable, highly needed genetic targets for increasing grain yields.

## Introduction

It is becoming increasingly difficult to make large increases in crop yield potentials for many reasons. Consequently, the crop yields likely to be achieved on current projections are inadequate to satisfy requirements (Ray *et al.*, 2013; Vos and Bellu, 2019). This is a serious situation. Failure to supply enough food, feed, and fiber would cause catastrophes throughout the world in food supplies, health, social stability, poverty, and basic costs of living, especially when the needs to use less land, climate change, and reduced use of fertilizers are considered. While there is every reason to expect rates of yield gain to increase as precision genotyping (Rimbert *et al.*, 2018) and phenotyping are increasingly adopted, as larger experimental populations are screened using unmanned aerial vehicles (UAVs) (Volpato *et al.*, 2021), and many innovations that shorten breeding cycles (Watson *et al.*, 2018) are exploited, such developments do not address what gene/biochemical pathway systems to target to achieve breakthroughs in rates of yield increase.

Why are rates of yield gain not accelerating given all the investments in plant science over the past few decades? There are many specific reasons. These include the large numbers of alleles [quantitative trait loci (QTLs)/haplotype segments] that need to be in a successful variety to achieve high yields, the comparatively low rates of incorporation of new alleles into some breeding programs, and desirable alleles being genetically linked to inferior alleles that are difficult to remove by recombination. Others are associated with the developmental complexities of spikes and of the grains within them. Spike development suffers from sensitivities to various stresses, including lack of nutrients, and many potential grains are lost through abortion (Sakuma and Schnurbusch, 2020). Current yields are often limited by inefficiencies within spike development causing yields to be ‘sink limited’. There is therefore a strong case for extending the numbers of known gene targets in spike development that can be exploited to drive yield improvements based on increasing the efficiencies of the many developmental processes and reducing interferences from stresses. This does not mean that improvements in ‘source strength’ which feeds spike development are not essential (Reynolds *et al.*, 2022; Slafer *et al.*, 2022). ‘Source strength’ is simply outside the scope of this review.

There are many examples which indicate that further increases in yield through changes in spike development are possible without leaving spike development vulnerable to increased stresses, floret abortion, and lower yield potential (Dreccer *et al.*, 2014; Wang *et al.*, 2017; Guo *et al.*, 2018a, c; Liu *et al.*, 2018; Lichthardt *et al.*, 2020). This review highlights a potential for exploiting systems based on the complexes complexes target of rapamycin complex 1 (TORC1)/sucrose non-fermenting-1 (SNF1)-related protein kinase, which drive growth and stress resilience, respectively, to use available nutrients more efficiently and maximize grain production in spike development. The contribution is also prompted by the need to find and exploit genes of high impact and value which are simpler to manage in breeding programs, just as dwarfing gene alleles enabled generation of the ‘Green Revolution’.

## Spike development: conflicts between growth, stresses, and competing sinks

Wheat yields are defined as the numbers and weight of grains per spike and numbers of spikes per square unit of land. Spike development, like all developmental processes with multiple meristems, is hugely complex. The correct balances of molecules feed into the inflorescence meristem and enable highly controlled cell divisions and subsequently cell expansion through biosynthesis of metabolites and polymers including cell walls. Meristems controlled by auxin, gibberellin, and cytokinin determine the body plan of the spike from read-out of their genomes via complex, epigenetically regulated chromatin architecture, transcription factors, RNA transcription and turnover, protein synthesis/turnover, metabolism, hormone action, secreted peptides, and patterns of cell division. Huge numbers of metabolites and energy-generating processes interact with these systems to achieve biosynthesis of macromolecules and metabolism in the right place and at the right time. Growth is also linked exquisitely into external and internal environments by signaling systems, some involving hormones, that facilitate these links. Thousands of genes promote and respond to cascades of developmental signals and processes to build the spike itself and the florets, and subsequently grains which form within it (Wang *et al.*, 2017; Li *et al.*, 2018). The genetic and physiological complexities underlying development present great challenges to breeders seeking to create plants that routinely generate more grains in agricultural environments because the number of genetic variants required for high yields is too great to manage.

**Fig. 1.**
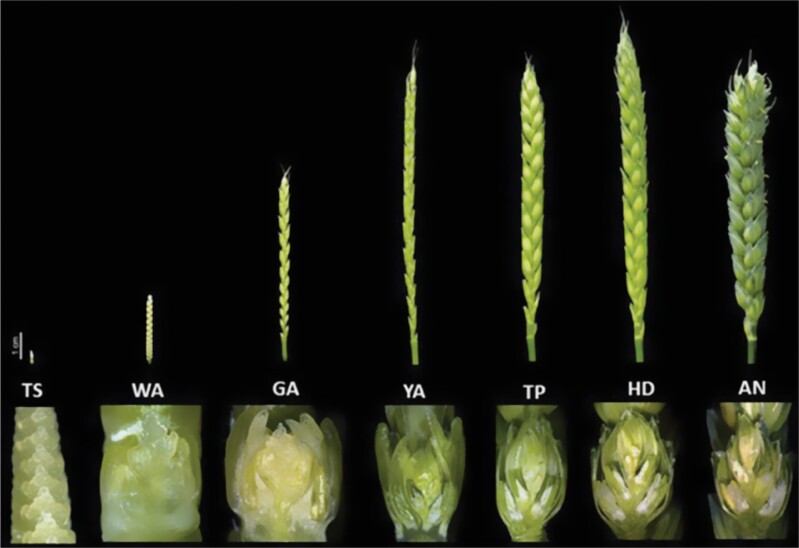
Spikes (top row) and spikelet sectional images (bottom row) at successive developmental stages: TS, terminal spikelet; WA, white anther; GA, green anther; YA, yellow anther; TP, tipping point; HD, heading; AN, anthesis. Spike length increases rapidly from TS to YA. Main accumulation of spike dry weight occurs from YA to AN. Scale bar=1 cm. Reproduced from Guo *et al.* (2018a). Floret abortion is readily detected in field-grown plants from GA (pre-meiosis) onwards, while in greenhouse-grown plants it was delayed, perhaps due to more stresses in the field. Abortions can continue to increase through to anthesis. There is significant variation in the timing and extent of abortion between varieties (Guo and Schnurbusch, 2015).

Spike development begins when the vegetative meristem of a stem becomes converted into the inflorescence meristem. Several key genes are known that determine the timing of this conversion (e.g. Vrn-1, Ppd-1, FT-1/Vrn-3 FT2; Kazan and Lyons, 2016; Gol *et al.*, 2017; Sakuma and Schnurbusch, 2020). The particular alleles of these genes that are deployed in agriculture match spike development to local daylength and temperatures to minimize resulting stresses and hence maximize the numbers of grains/spikes. The inflorescence meristem programs spike growth. Spikelet meristems emerge to form branch-like spikelets until the terminal spikelet is formed. The stages of spike development from terminal spikelet formation to anthesis are illustrated in Fig. 1 (Guo *et al.*, 2018a). The spikelet meristem is indeterminate and so many florets are established within each spikelet. This indeterminate feature is the origin of the many florets which do not develop fully but abort later in development, before anthesis (Fig. 1). The number of spikelets which are laid down in the inflorescence is genetically, spatially, and environmentally determined (Dreccer *et al.*, 2014; Wang *et al.*, 2017; Guo *et al.*, 2018a, b; Liu *et al.*, 2018; Backhaus *et al.*, 2022; Slafer *et al.*, 2022) and also relates to the rate and duration of spike development. Each floret develops from the floret meristem, generating two bract-like structures, the lemma and palea, three anthers (stamens), two lodicules, and one carpel. Cells within the carpel and stamen proceed through a long pre-meiotic cell cycle with DNA replication and then through meiosis proper to form gametes. Anthers dehisce in the floret, release the pollen, and ovule fertilization occurs. Anthers then emerge and residual pollen is shed. There is a large increase in spike length and weight between when the terminal spikelet is formed and anthesis (Fig. 1) that leads to competition for resources between the florets and other parts of the spike—an inbuilt source of stress. The associations between floret abortion, stem length, and spike dry matter growth support the hypothesis that floret abortion is mainly due to competition between florets, spikes, and stems for nutrients (see Fig. 1) (Fischer, 2011; Gonzalez *et al.*, 2011; Gol *et al.*, 2017; Guo *et al.*, 2018a; Pretini *et al.*, 2021; Slafer *et al.*, 2022).

The abortion of some 60% of the florets (Sakuma and Schnurbusch, 2020) is a huge loss of yield potential and a major reason why yields are not higher. This abortion needs to be reduced substantially to increase fruiting efficiency—grains per gram of spike dry weight at anthesis (Pretini *et al.*, 2021). However, the existence of excess florets means that a potential route to achieving major additional gains in wheat yield potential is already wired into the organ developmental program. Many examples from breeding and research programs have, over the years, illustrated increases in yield through complex genetic selections that overcome some floret abortions (Pretini *et al.*, 2021; Slafer *et al.*, 2022). Also, a single gene regulating the numbers of aborted florets and fruiting efficiency has been uncovered. (Sakuma *et al.*, 2019). This gene, *GNI 1*, encodes a transcription factor which inhibits floral development and fertilization and has been proven to be valuable commercially. Reduced expression of the gene leads to enhanced numbers of grains per spikelet and hence grains per spike (yield). There is clearly a need for many genetic variants that achieve these traits to be found, or created, and to be validated in elite germplasm under agricultural conditions so that they can become targets for mutation and selection in breeding programs around the world (e.g. Wang *et al.*, 2017; Thirulogachandar *et al.*, 2021, Preprint; Backhaus *et al.*, 2022).

A large number of publications over many decades have shown that almost any kind of stress can result in fewer florets reaching maturity and aborting before fertilization (see, for example, Gol *et al.*, 2017). The stages of spike development between spikelet formation and anthesis are especially sensitive to stresses, as noted in Fig. 1. This includes the stages in pre-meiosis, taking place before/around the ‘green anther stage’ (Fig. 1) (Kirby, 1988) that are sensitive to higher temperatures (Draeger and Moore, 2017). Breeders have not yet been able to select lines that are adequately resistant to such stresses. Therefore, both the yield potential and the yields formed in farmers’ fields are routinely compromised.

Crops suffer from many sources of stress that reduce fitness. Some are generated externally and others internally. The well-known externally imposed stresses come from drought, heat, cold, wind, high and low light, diurnal cycles, poor soil structures, lack of nitrogen and phosphorus, high density planting, toxic chemicals, and, of course, the many diseases that are not overcome by the genotype. Root-associated microorganisms and endophytes also play important parts in stress tolerance (Compant *et al.*, 2010).

External stresses become converted into internal stress responses and suboptimal biochemistry, physiology, and development. In any given location, some stresses are more important/relevant than others. Many factors belonging to stress biology play major roles in meristem development (Beveridge *et al.*, 2007). For example, meristems are under the stresses of internal pressure (Beauzamy *et al.*, 2015). Reactive oxygen species (ROS) are sources of stress but are also vital signaling molecules that shape meristems and stem cell identity, and contribute to the regulation of cell division and organogenesis (Huang *et al.*, 2019). The process of flowering and its timing are also driven by stress signaling molecules, and the major alleles exploited to minimize the effects of stresses are well known, as reviewed by Sakuma and Schnurbusch (2020); Kazan and Lyons (2016) and Gol *et al.* (2017). Perhaps the most frequent and consequential stresses in spike development are those created by inadequate amounts of nutrients being transported from the leaves and stem into the spike (Ghiglione *et al.*, 2008). Such limitations can be caused by lack of photosynthesis, failure to convert stored carbohydrates to sugars, and inadequate transport of sugars, N, P, and S, etc. into spikes versus other plant organs. It is noteworthy that the dwarfing genes that gave rise to the ‘Green Revolution’ result in increased amounts of nutrients flowing into the spike, thereby relieving limitations (Reynolds *et al.*, 2022). Internally generated stresses may also be created by some, especially distal, florets being inadequately linked to the vascular systems that supply nutrients and water (Whingwiri and Stern, 1982).

The presence of stresses within developing spikes has also been inferred from RNAseq analyses of spikes harvested at different stages, from formation of the inflorescence meristem until anthesis (Wang *et al.*, 2017; Li *et al.*, 2018; Thiel *et al.*, 2021). Transcripts encoding proteins and long non-coding RNAs (Cao *et al.*, 2021) associated with stress resistance are expressed throughout spike development but particularly at stages of spikelet formation and beyond. It appears that the long non-coding RNAs are mainly associated with stress and wound responses in addition to transcription (Cao *et al.*, 2021). All these RNAs clearly indicate the presence of stresses and systems of stress management, but their precise locations and those of the proteins they specify are as yet unknown. They may be concentrated in the florets most likely disposed to abort and not in the florets most likely to develop viable gametes (Thiel *et al.*, 2021). Floret abortion results from an active, stress-induced program. Subsequent autophagy (Ghiglione *et al.*, 2008) may have been selected here to recycle the molecules to feed the remaining viable florets, in which case stopping development of some florets may be an active process to support survival of others. The conclusions that spikes not deliberately treated with an external stress have at least some cells/florets already under stress, undergoing autophagy, and where abscisic acid (ABA) is localized (Boussora *et al.*, 2019; Zhang *et al.*, 2019; Thiel *et al.*, 2021) suggest the hypothesis that spikes and especially (some) florets are exceptionally sensitive to external stresses because they are already suffering from aspects of internal stress biology and autophagy.

As florets and spikes mature, photosynthetic systems are induced and the organ becomes a source of fixed carbon, useful for ongoing floret and spike growth. This can presumably alleviate any shortages coming from the leaves and stems as they start to senesce. Thus, the sooner spike photosynthetic competence, linked to internal vascular systems, is achieved the greater the likelihood that more florets will survive and there is an adequate supply of assimilate for grain filling after anthesis.

After fertilization, embryo and endosperm growth occurs, with endosperm featuring endoreduplication cycles without cell division to form a syncytium. Cellularization of the endosperm then occurs followed by grain filling via starch and protein biosynthesis. The final phase of grain development involves shutdown of metabolic activities, programmed cell death, desiccation, and induction of dormancy (Shewry *et al.*, 2009, 2012). The process of grain filling determines the final thousand grain weight. Cytokinins and ABA control the induction of dormancy and its maintenance (Tuan *et al.*, 2019).

## Control of growth and stress by protein phosphorylation and dephosphorylation

Cells sensing a stress typically reduce or stop biosynthetic pathways associated with growth, unless the biosynthesis is necessary for stress resistance, and then adjust to the stress condition by activating stress resistance metabolic pathways driven by a different set of genes and regulatory molecules. When the stress has passed, they may switch back. Switches can occur rapidly, whereas readjustment of metabolism via changes in siRNAs, epigenetics, gene transcription, protein synthesis, etc., while vital for phenotype manifestation, usually takes much longer. Therefore, the switching systems and the signal transduction systems associated with them are very important parts of protection systems, such as those needed in the rapid phases of spike development where stress conditions can arise rapidly. Switches and signal transduction systems often involve protein phosphorylation and dephosphorylation, making kinases and phosphatases key rapid regulators of growth and stress resilience in organisms.

Large numbers of protein kinase and phosphatase genes serve as regulators in plants (Chen *et al.*, 2021; Lin *et al.*, 2021). Protein kinase genes were found to make up ~5.5% of the Arabidopsis genome (Schulze, 2010). Many sites in proteins are predicted to be substrates for phosphorylation, but only a fraction of these have been characterized to date (Van Leene *et al.*, 2019). In a large-scale phosphoproteome study in Arabidopsis, some 5300 phosphopeptides were identified, representing 2500 unique phosphoproteins (Wang *et al.*, 2013), while in a smaller study on wheat some 1200 phosphoproteins were defined including many groups of transcription factors (Zhang *et al.*, 2014). Most had one phosphorylation site but some had up to three sites. As expected, most were phosphorylated at serine or threonine resides but a few at tyrosine residues. Examples of networks of phosphoproteins, protein kinases, and phosphatases that control growth and development were described, including those responsible for managing the plant’s response to stresses. Some 200 phosphoproteins were found to undergo phosphorylation changes in response to drought, and variants between varieties that differed in drought tolerance were classified. Many of these proteins were involved in RNA transcription/processing, stress detoxification/defense, and signal transduction (Zhang *et al.*, 2014).

At the top of the regulatory phosphorylation chains regulating and coordinating growth and stress resilience are the TORC1 and SnRKs 1, 2, and 3 which are master regulatory kinases. As described below, these protein complexes are regulated to reciprocally control each other, resulting in a trade-off between growth and stress control. The central concept of trade-off is particularly important to this review because spike development and increasing grain production potential clearly involve both rapid meristematic and cell growth and management of internal and external stresses, as described above. Because spike development is rapid, there is probably little time for recovery once stress has interfered with a developmental process. The result in the case of florets is abortion. Given that the central goal of plant breeding is to create high yielding plants tolerant to many stresses, these phosphorylation systems are worthy of much attention but have not received focused attention in crop breeding programs to date. They are likely to be where breakthroughs in optimizing yield gains and spike resilience can be found, engineered, or selected.

## The TOR–SnRK control systems behind the growth–stress trade-off in spikes

The master complexes at the heart of the growth/stress control systems are the kinase complexes: TOR and SnRK1, SnRK2, and SnRK3. TOR plays a central role by integrating nutrient, energy, hormone, and environmental inputs to program cell proliferation, growth, and metabolism. SnRK proteins and their controlling mitogen-activating protein kinase kinase kinases (MAPKKKs) (Pizzio and Rodriguez, 2022) sense stresses, energy deprivation, and nitrogen and phosphorus deprivation, and reprogram metabolism to preserve survival by inducing stress resistance pathways. These kinase systems work together in intricate ways, both in concert and antagonistically, to achieve the balances and trade-offs required for rational growth and survival under different environmental conditions and in different cell types, tissues, and organs. Most, but certainly not all, of the discoveries in plants have been made with Arabidopsis but, given the conservation of the SnRK and TOR systems across kingdoms, it is reasonable to assert that the principles established via Arabidopsis and other plant species are very relevant to wheat (Shi *et al.*, 2018; Mao *et al.*, 2020).

### TOR

TOR occurs in the complex TORC1 whose three core components are TOR, RAPTOR (a regulatory protein), and LST8 (a small associated protein). There may be active forms in which one or more proteins of the complex are absent. Dimers of the TORC1 complex may also be formed in certain situations. TOR complexes regulate cell division rates and are expressed highly in rapidly growing tissues, such as the embryo, and shoot and root meristems, where cell proliferation occurs (Shi *et al.*, 2018; Wang *et al.*, 2018; Wu *et al.*, 2019). In addition, TOR signaling is involved in adult plant development through leaf expansion, flowering, shoot branching, and senescence. Photosynthesis-derived glucose and sucrose are potent activators of TOR (Li *et al.*, 2017; Margalha *et al.*, 2019). The growth-promoting hormone auxin also stimulates TOR activity through a physical interaction with an auxin-activated GTPase (Li *et al.*, 2017). TOR also mediates the crosstalk between sugar signaling and brassinosteroid/cytokinin signaling to stimulate hormone-controlled growth (Z. Zhang *et al.*, 2016; Janocha *et al.*, 2022, Preprint).

TOR also acts as a master regulator of photosynthesis (Dong *et al.*, 2015) and of gene expression by coordinating rRNA transcription, ribosomal protein gene activation, ribosome assembly, and translational control (Sesma *et al.*, 2017; Schepetilnikov and Ryabova, 2018) including transcription and translation of plastidic ribosomal proteins (Dobrenel *et al.*, 2016). It dictates transcription reprogramming (Fu *et al.*, 2021) of diverse sets of genes involved in central and secondary metabolism, the cell cycle, transcription, signaling, transport, and protein folding. While TOR signaling is induced by nutrients, various growth factors, and light, it is inactivated and down-regulated by energy deprivation, starvation, and multiple stresses (see below). These very wide-ranging properties of the TORC1 complex reveal its central contribution to the regulation of plant development, crop phenotypes, and yield traits.

### SnRK genes

There are many genes specifying SnRKs in plants (Margalha *et al.*, 2019). Mishra *et al.* (2021) reported that for hexaploid wheat there are 14 SnRK1, 65 SnRK2, and 95 SnRK3 genes. SnRK genes are differentially expressed within and between groups in different organs, including spikes, and in response to different stresses, including pathogens, but some redundancies are likely within the family (Mishra *et al.*, 2021). SnRKs function as heterotrimeric complexes composed of one catalytic subunit (α) and two regulatory (β- and γ-type) subunits (Crepin and Rolland, 2019). They are central energy sensor and transcriptional regulators that program plants to respond to stresses, carbon and energy, and nitrogen and phosphate deficiencies, and restore homeostasis (Mao *et al.*, 2010; Nukarinen *et al.*, 2016). There are cytosolic and nuclear complexes, with movement between compartments being part of the regulatory systems. SnRKs orchestrate a broad transcriptional reprogramming of down-regulation of anabolism and up-regulation of catabolism, inducing cell wall, starch, protein, and lipid degradation partly through direct phosphorylation of metabolic and regulatory proteins and partly through phosphorylation-driven changes to key transcription factors (Crepin and Rolland, 2019; Margalha *et al.*, 2019; Mao *et al.*, 2020). Over 1000 SnRK1-regulated proteins were identified in Arabidopsis following overexpression/knockout of an SnRK1 gene in mesophyll protoplasts (Nukarinen *et al.*, 2016).

SnRK1 activity is under complex regulation, and SnRK2 family members respond to multiple types of stress such as water deficit, salt, and cold to confer stress resistance, and individual members have acquired distinct regulatory properties (Nukarinen *et al.*, 2016; H. Zhang *et al.*, 2016; Mao *et al.*, 2020). SnRK2s stimulate stress resistance responses through complex networks of signal transduction via both ABA-dependent and ABA-independent resistance mechanisms. [ABA is a hormone formed in response to many stresses, especially drought (Ma *et al.*, 2018; Islam *et al.*, 2021)]. The regulatory linkages between ABA, SnRKs, and downstream activities are multiple and complex, involving ABA receptors and phosphatases. They are also controlled positively or negatively by the phosphorylation of SnRKs by B2, B3, or B4 RAF-like or C-type MAPKKKs (Lozano-Juste *et al.*, 2020; Kamiyama *et al.*, 2021a, b; Sun *et al.*, 2022). The promoters of wheat *TaSnRK2* genes carry a large number of stress regulatory *cis*-elements predicted to determine their patterns of activation responses to different stresses. Most *TaSnRK2* genes are highly sensitive to osmotic stress because their transcription is strongly induced within an hour of osmotic stress.

SnRK1 is inhibited by trehalose 6-phosphate (T-6-P), a key regulatory signaling molecule (Paul *et al.*, 2020), glucose 6-phosphate (G-6-P), and glucose 1-phosphate as part of the carbon-sensing system (Nunes *et al.*, 2013), and phosphorylates trehalose phosphate synthase (TPS), fructose-6-phosphate, 2-kinase/fructose-2,6-bisphosphatase, and ADP glucose pyrophosphorylase to control their activities (Kulma *et al.*, 2004). The SnRKs also phosphorylate and inactivate other important growth-promoting enzymes, such as 3-hydroxy-3-methylglutaryl-coenzyme A reductase, sucrose phosphate synthase, and nitrate reductase (Sugden *et al.*, 1999) to control metabolism. SnRK3 genes probably evolved from SnRK2 genes. They provide additional diversity for regulating phosphorylation of proteins in different cell types and in response to different stresses. The potential diversity of responses involving combinations of SnRK1, 2, and 3 is large, providing therefore a huge potential for optimally directing all the stress and growth-determining enzymes, transcription factors, and metabolic pathways to keep cells optimally tuned to their local environments. They undoubtedly play a major role in crop performance, including in spike development, by programming and regulating survival pathways and also inhibiting growth in response to biotic and abiotic stresses. TOR activity is also controlled by phosphorylation via SnRK1 in association with SnRK2 under conditions of energy deprivation and in the absence of T-6-P inhibition (see below). While in many situations SnRKs and TOR are regulated in opposite directions, there are circumstances where they are both induced. This could be because they are present in different cells, responding to different local environments, or, because, for example, localized growth is required as part of the defense mechanisms. (Margalha *et al.*, 2019).

## The molecular interactions between TOR and SnRKs that produce the stress resilience–growth trade-off in spike development

**Fig. 2.**
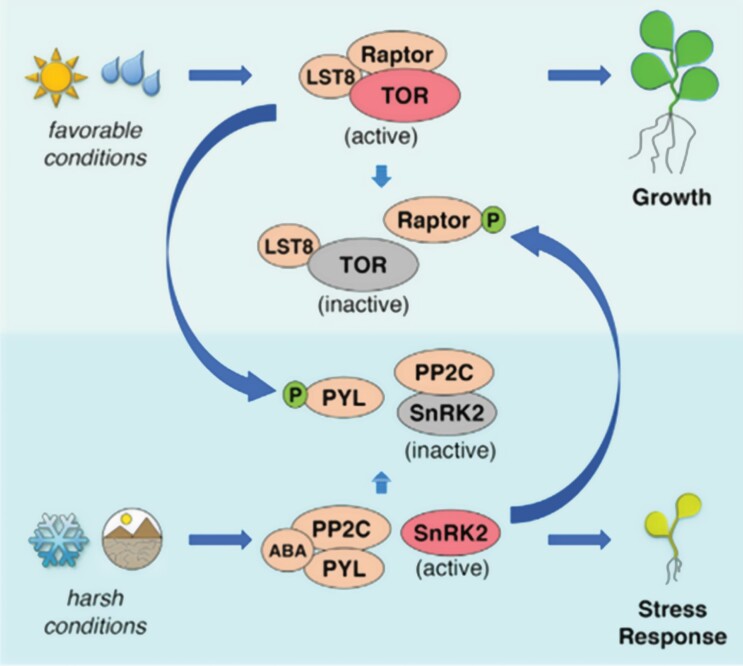
Scheme illustrating controls of growth and stress responses by interacting TOR and SnRK complexes. Under favorable conditions, the TORC1 kinase, comprising TOR, Raptor, and LST8 proteins, promotes growth and inhibits the stress response by phosphorylating the ABA receptor PYL that subsequently inhibits SnRK2 via the pyrophosphorylase 2C (PP2C). C-type MAPKKKs also negatively regulate ABA-activated SnRK2s under favorable growth conditions to maintain active TOR (Pizzio and Rodriguez, 2022). Under stress conditions ABA binds to PYL and sequesters PPC2 which leads to SnRK2 activation and cellular stress mitigation responses, with B-type MAPKKKs being involved in the phosphorylation and activation of SnRK2s (Pizzio and Rodriguez, 2022). Active SnRK2 molecules then phosphorylate Raptor which causes the dissociation of TORC1 and inhibition of growth. [Reproduced with permission from Rosenberger and Chen, 2018), Copyright Elsevier (2017).]

The ‘trade-off’ interactions between TOR and SnRK activities that orchestrate plant development under prevailing external and internal environments are multiple and complex (Henriques *et al.*, 2014; Z. Zhang *et al.*, 2016; Baena-Gonzalez and Hanson, 2017; Margalha *et al.*, 2019; Kamiyama *et al.*, 2021a, b). Understanding of the molecular mechanisms behind the trade-off have emerged over the past decade. One exemplifying model built from extensive studies in Arabidopsis is illustrated in Fig. 2.

In the presence of stresses, such as drought, cold temperature, or nutrient starvation, ABA is produced and moves through the plant (Rosenberger and Chen, 2018; Wang *et al.*, 2018). It binds to a receptor PYL and sequesters pyrophosphatase 2C (PP2C), thereby enabling activation of SnRK2 by MAPKKK phosphorylation (Lozano-Juste *et al.*, 2020) and also enabling SnRK2 to perform the range of kinase activities outlined above to promote downstream stress resilience pathways and networks. Activated SnRK2 also phosphorylates Raptor proteins, causing the loss of Raptor from the TORC1 complex, thereby preventing TORC1’s growth-promoting activities. These reactions therefore both activate stress resilience metabolism and inhibit growth-promoting systems. When stress is absent, sugars are available promoting TOR to be active, and T-6-P inhibits SnRK1, thereby stopping induction of the SnRK1-stimulated stress-induced pathways. TOR can then phosphorylate the ABA receptors to prevent activation of the stress responses via ABA, thereby enabling TORC1 to promote the growth pathways. These reciprocal phosphorylation loops between the ABA core signaling components, SnRK2 and the TOR complex, result in the balance/trade-off between stress resilience and growth, and enable rapid adaptation to continuously changing conditions (Wang *et al.*, 2018).

The quantitative consequences of local trade-offs (growth or resilience or states in between) presumably reflect the TORC1/SnRK activity ratios in specific cells together with the molecules and many downstream pathways/networks they control via phosphorylation, dephosphorylation, and other mechanisms. For example, very different TOR/SnRK activity ratios are likely to be present in young, actively maturing florets compared with those destined to abort.

**Fig. 3.**
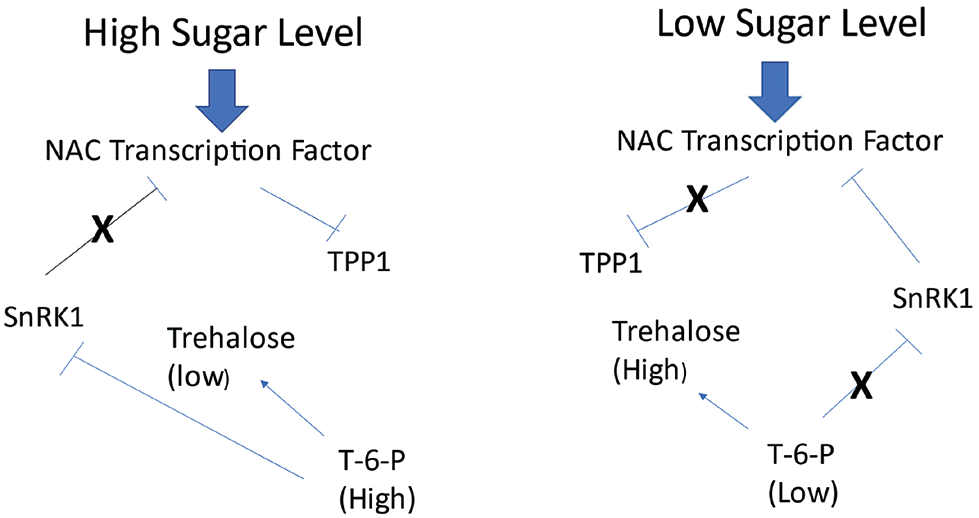
Model for the role of T-6-P and its control by a sucrose-inducible transcription factor in response to sugar levels. Under high sugar, the NAC transcription factor is induced which down-regulates TPP1 leading to increased levels of T-6-P. This blocks SnRK1 activity and reduces inhibitory feedback on the NAC transcription factor, thus driving the cycle even higher. Under low sugar, induction of the NAC transcription factor is reduced, resulting in more TPP1 and conversion of T-6-P to trehalose. The reduced amounts of T-6-P allow SnRK to remain active and inhibit any NAC transcription factor molecules. This cycle therefore drives low sugar levels and loss of growth. Schemes are from Li *et al.* (2022). T-6-P, trehalose-6-phosphate; TPP1, trehalose phosphate phosphatase 1 which converts T-6-P into trehalose.

Additional roles of T-6-P and its regulation fit into these scenarios. T-6-P is made from UDP-glucose and G-6-P via TPSs and is converted into trehalose by trehalose phosphate phosphatases (TPPs). As noted later, there are many differentially regulated variants of these enzymes in wheat and other plants. In rice, a sugar-inducible transcription factor represses the transcription of certain TPPs that consequently leads to increased levels of T-6-P, inhibition of SnRK1 activity, and increased TOR activity, probably via increased sucrose levels (Li *et al.*, 2022). These in turn release repression and translation inhibition of the transcription factor by SnRK1, thereby promoting even higher levels of T-6-P and growth. This is illustrated in Fig. 3. T-6-P is therefore another key regulated molecule involved in the central control of growth versus inhibition of growth (Figueroa and Lunn, 2016; Li *et al.*, 2022). Furthermore, in maize, TPP also serves as a regulator of differentiation of inflorescence meristems, but this suppressing role appears not to require its enzyme activity (Demesa-Arevalo *et al.*, 2021, Preprint).

## Spike development, TOR/SnRK balance, and ‘sink strength’

As noted above, the concentration of active TOR and the TOR/SnRK activity ratios are major determinants of rates of growth in meristems and differentiated cells by being the activators of large numbers of downstream cascades of pathways and networks that achieve growth and organ traits. This leads to the following hypotheses and high-level framework for the regulation of spike and grain development.

The amounts and activities of TOR are determined by concentrations of local (signaling) inducers: sugars, T-6-P, auxin, cytokinin, nitrogen, and phosphorus. Because there is little light within the stem where spike growth is initiated, the concentrations of nutrients, T-6-P, and auxin are likely to be the dominant inducers. As spike development continues through successive formations of lateral meristems, spikelets, and florets, the activities of TOR versus SnRKs continue to determine rates of growth of each component and overall spike growth. A continuing supply of inducers and nutrients is essential for sustaining high TOR/SnRK ratios and growth. The higher the rates of supply to meristems, the larger the number of activated TOR molecules, the higher the rates of activation of protein synthesis and downstream biosynthetic pathways, and the greater the biomass and numbers of spikelets, florets, and spike mass formed, as supported by extensive experimental evidence (e.g. Gonzales *et al.*, 2011; Pretini *et al.*, 2021). Where florets are less well served with inducers of TOR, or by the vascular systems, or suffer higher levels of stress, unfavorable TOR/SnRK ratios form in critical cells. Such florets struggle to develop into fully fertile florets and, eventually, reach a point of no return. Their cells with low TOR/SnRK ratios switch from the TORC1-led pathways to the SnRK-led pathways, resulting in low or no growth. Autophagy driven by SnRKs is then initiated. When florets and subsequently ovules have low concentrations of TOR and low TOR/SnRK ratios compared with other parts of the spike, overall spike but not floret growth is favored.

Grain filling, especially the synthesis of starch and protein bodies in the endosperm, is a very important phase underpinning yield. High rates of biosynthesis are required, supported by availability of sugars, nitrogen, and other nutrients. The early phase of grain filling is accompanied by very high levels of T-6-P in filial tissues, including the endosperm, which correlates with amounts of sucrose and induction of TOR, which is likely to be highly active. The high levels of T-6-P inhibit the SnRKs present to ensure the high rates of biosynthesis of starch and protein in the endosperm (Martinez-Barajas *et al.*, 2011). These results emphasize that the activity ratio of TOR to SnRKs is determined not only by amounts of the proteins but also by the concentrations of inducers and inhibitors in the relevant cells and also their phosphorylation state (Coella and Martinez-Barajas, 2014). The final phase of grain development involves shutdown of metabolic activities, programmed cell death, desiccation, induction of dormancy (Shewry *et al.*, 2009, 2012), and high levels of stress-induced proteins, many of which are phosphorylated (Ma *et al.*, 2014). The process of grain filling determines the final thousand grain weight. Cytokinins and ABA control the induction of dormancy and its maintenance (Nakashima *et al.*, 2009; Tuan *et al.*, 2019). TOR is expected to be suppressed and inhibited under the conditions of desiccation and ABA-induced stress physiology.

While high rates of phosphorylation/dephosphorylation cascades initiated from TOR are important, growth and organ traits can also be promoted by additional genetic/physiological interventions in downstream pathways and networks to overcome any early developmental, environmental, or spatial shortcomings in organ promotion not fully achieved by TOR stimulation. This has been demonstrated by, for example, Wang *et al.* (2017), Thirulogachandar *et al.* (2021, Preprint), and Backhaus *et al.* (2022) who analyzed transcription profiles at different stages of spike development, nominated candidate transcription factors, and showed that changing their expression led to changes in sizes of spikes and grain numbers per plant. The extent to which activation of these downstream pathways involved changes in phosphorylation states is not known.

The above framework based on known properties of TOR implies that the activities of TOR, determined by levels of inducers and TOR/SnRK activity ratios in spikelets and florets, and their downstream pathways are major determinants of ‘sink strength’. ‘Sink strength’ determines the numbers and size of grains formed within the spike. Plants with high ‘sink strength’ (TOR-driven activities) have the ability to assimilate larger amounts of nutrients into their spikelets and florets. The biomass of the grain relative to the whole-plant biomass, including the non-grain parts of the spike, is termed the ‘harvest index’. This hypothesis therefore implies that TORC1 activities, TOR/SnRK activity ratios, and regulation of their downstream pathways are also the major determinants of the ‘harvest index’.

Because the amounts and ratios of TOR/SnRK molecules determine rates of growth differentially during the phases of spike development, it would be expected that there would be genetic relationships between yield potential and rates of spike growth at different stages of development. These have been found (e.g. Guo *et al.*, 2016a, b; Liu *et al.*, 2018; Li *et al.*, 2021). Associations between plant biomass and yield potential are also predicted, as has been found (Guo *et al.*, 2016a). Where there have been increases in yield without increases in total plant biomass, as found in some breeding programs in recent years (Mondal *et al.*, 2020), breeders have presumably selected variation that affects inducer concentrations, TOR and/or TOR/SnRK ratios in the spikelets and florets but not in other vegetative meristems and cells, or at least not to the same extent.

## TORC1, SnRKs, and plant breeding

The knowledge that TOR and SnRKs control each other, resulting in reciprocal trade-offs of growth and stress resilience, provides an explanation for why it is difficult for plant breeders to readily select lines that have both high grain production and high resilience of spike development to stresses. The difficulties are compounded when the developmental process has inbuilt generation of stresses or inadequate supplies of nutrients from the stem and leaves. Breeders have to select for both yield and stress resilience. Selection for only one is very unlikely to create a commercially successful variety. This is one reason why yield gains are made relatively slowly today. Any selected trade-off is likely to be one of many that could have been achieved to provide a similar overall result and not likely to be confined to any single pathway or trait/subtrait. Given this complexity, focusing on genetic variation at or near the top of the phosphorylation regulatory systems, with its simpler genetic basis, appears attractive in searches to boost grain production.

The central hypothesis of this review suggests that control of the large numbers of cascades of events in spike and floret growth controlled by phosphorylation/dephosphorylation switches is not optimal but balanced too much in favor of overall spike growth rather than spikelet and floret growth and/or too much in favor of growth inhibition when stresses emerge, also resulting in insufficient floret developmental growth. The experimental approaches for improving grain yields described below therefore seek to increase the TOR/SnRK activity ratios in spikelets/florets during spike development. Expected outcomes based on known functions of TOR and SnRKs and spike development would include (i) greater number of active TOR molecules in key cells, (ii) a more favorable competitive balance of nutrients between spikelets/florets and other spike parts, and (iii) more rapid cell divisions leading to stronger spikelets and florets with greater floret survival and fruiting efficiency. If successful, the resulting higher ‘sink strength’ could require a greater ‘source strength’ to maximize yields, but new technical approaches to achieve this are outside the scope of this particular review (See Murchie *et al.*, 2022).

### Changing TOR and SnRK activities to achieve gains in yield

Genetic mutation studies in other species show that it is possible to change the balance and trade-offs between growth and stress tolerances. Given the large numbers of SnRK genes in wheat and the need to drive spike development more rapidly, it would appear more straightforward to alter TOR gene activities, noting that the TORC1 complex contains two additional proteins. Also, although it might seem appealing to boost stress resilience genes during spike development to overcome stresses, if this results in loss of growth through trade-off then the purpose would be defeated. Several approaches, and combinations of approaches, should be explored based on the central hypothesis of this review to change the spike growth/stress resilience/location balance in desirable ways. Some of these are outlined below.

#### Screening germplasm for plants with altered levels of expression of TOR and SnRK genes in spikes

A Korean wheat line, Iksan 370, which has a giant phenotype in terms of whole-body architecture, being taller and having longer leaves and spikelets, was found to have highly elevated levels of TOR (Shin *et al.*, 2015). It has larger leaf epidermal cells resulting in larger leaf architecture, but photosynthesis did not differ from a control. The line also showed higher yields than a reference line from the same breeding program (Kim *et al.*, 2019). TOR activity in spikes was not noted. These reports imply that breeders can select TOR variants either via specific phenotypes or by screening TOR transcript levels in specific organs to find altered TOR/SnRK expression ratios. Such alleles can be then tested for their value. Cultivar releases over time from breeding programs could also be examined for TOR/SnRK activity ratios to explore how the ratios have been changed over time in response to breeding and selection.

#### Altering amounts of TOR via transgenesis and/or genome editing

This approach has already been successfully demonstrated in rice (Bakshi *et al.*, 2017). Ectopic expression of Arabidopsis TOR cDNA, under the control of the 35S promoter, which programs activity in most types of cells, was associated with up-regulation of OsRAPTOR and OsLST8 in greenhouse-grown plants. The plants had increased tillering, plant height, panicle length, and leaf area, and higher Chl *a* and *b* in leaves, as would be predicted. Height correlated with levels of AtTOR expression. High expressors also had high water use efficiency (biomass produced per unit of water transpired by the crop), more lateral roots, and higher levels of specific osmotic protection and drought-responsive genes. This example illustrates the ability to change the phenotypic trade-off by incorporating a foreign TOR under the control of a different promoter and selecting suitable levels of transgene expression.

In spite of the interesting leads discussed above and given the expected wide range of effects of changing the expression of TOR/SnRK genes, a better approach is likely to be based on leaving the existing, highly selected composition of TOR/SnRK genes found in elite lines and adding a TOR gene to an elite chassis with a promoter that restricts additional TOR activity to just those phases (i.e. early spikelet and floret initiation and development where more active growth is required; see Paul *et al.*, 2018b). Such promoters can be selected from analyses of the gene expression profiles during spike development (Wang *et al.*, 2017; Li *et al.*, 2018; Thiel *et al.*, 2021). This would shift the growth balance at the specific time and in the specific organ, thereby reducing the probability of generating unwanted phenotypes including larger spike non-grain biomass. Such a shift in TOR activity should not, for example, cause major upsets for good canopy production or vegetative growth. If addition of such alleles creates unwanted phenotypic variation, this can be selected against via modification of transgene expression or in a breeding program designed to vary the genetic background to achieve the desired phenotype.

Although especially targeted transgenes appear attractive, as mentioned above, they are not ideal for crop varieties for many reasons, including that they often become methylated and lose activity (Anand *et al.*, 2003). It also needs to be noted that control of expression is often complex due to activities of siRNA, non-coding RNAs, and gene localization, and these phenomena can undermine promoter choice and stable transgene expression. However, if it were possible to edit (Mao *et al.*, 2019) the promoter of one of the existing TOR genes to provide the desired pattern of expression, as described above, then the equivalent results may be obtained, so generating the required shift in TOR activity ratios.

#### Seeking genetic variation around TOR/SnRK loci that is correlated with important traits

Given the large number of SnRKs in determining stress resilience and yield, it can be predicted that particular combinations of variants of the ~170 SnRK genes are positively selected in wheat breeding, together with variants of the huge numbers of proteins phosphorylated by these kinases during spike development in the external and internal environments encountered during crop growth. Many existing QTLs and loci discovered via genomic selection may therefore align with TOR/SnRK genes, thus revealing potentially useful genes. This has been explored by Ur Rehman *et al.* (2019). The wheat protein kinase TaSnRK 2.9-5A gene was found to be associated with yield-contributing traits and located on chromosomes 5A, B, and D. Polymorphisms were found for the gene on chromosome 5A and associated with improved traits, and were used in cultivars released around the world. One allele, Hap 5A-1/2, was associated with thousand kernel weight (TKW) and has been selected in Chinese, Pakistani, East European, and West European breeding programs. Another allele, Hap-5A-4, had significantly higher grains per spike compared with other haplotypes. Rice lines overexpressing the allele 2.9-5A showed higher grains per spike, similar TKW, slightly reduced height, but slightly increased spike length. (Ur Rehman *et al.*, 2019). This example illustrates ways of finding alleles within the large multigene families and their crop value based on their association with traits and patterns of selection in breeding programs. These alleles are likely to influence the SnRK/TOR balance in certain cells/organs.

#### Changing SnRK activities by targeted transgenesis or editing

The expression profiles of >170 SnRK genes within organs and in response to stresses in wheat are complex (H. Zhang *et al.*, 2016; Mishra *et al.*, 2021). A specific subset is induced or down-regulated in spikes, and expression of some is probably controlled by miRNAs (Mishra *et al.*, 2021). From the expression results already available and the results noted above, specific SnRK genes could be selected and their expression levels increased transgenically or by editing, or reduced by RNAi or by editing (Mao *et al.*, 2019) to alter SnRK/TOR activity ratios and growth phenotypes in specific organs/times during spike development to modulate yield. For example, SnRK expression could be increased in non-floret cells to change the balance between floret and main spike growth in favor of florets. Alternatively, SnRK expression could be reduced in florets specifically. Noteworthy is the demonstration that overexpression of a wheat SnRK gene in Arabidopsis increased tolerance to drought, salt, and low temperature (Mao *et al.*, 2010).

#### Modifying TOR/SnRK activities by modulating and activating inhibitors

There is extensive evidence to indicate that modifying the concentrations of activators and inhibitors of TOR/SnRKs is a relevant way to modify the SnRK/TOR ratios, and this has undoubtedly been under selection in breeding programs. The supply of sucrose molecules from the stem into the inflorescence meristem and spike is an obvious example. The example of T-6-P in wheat is also particularly relevant (Lawlor and Paul, 2014). T-6-P inhibits SnRK1 (Fig. 3) and is related to levels of sucrose, an inducer of TOR, in many tissues. T-6-P is made from UDP-glucose and G-6-P via TPS and is converted into trehalose by TPP. Variation in these enzymes in amounts, spatial distribution, and activities would be expected to lead to variation in SnRK and TOR activities (Nuccio *et al.*, 2015; Paul *et al.*, 2018a; Li *et al.*, 2022). In an extensive study in elite germplasm, variation amongst the 25 TPS gene and 31 TPP genes in wheat has been shown to be under selection, separately and in combination, and associated with important yield-related traits (Paul *et al.*, 2020; Lyra *et al.*, 2021; Du *et al.*, 2022; see also Zhang *et al.*, 2017). Therefore, selection of specific variants known to result in higher yields via T-6-P levels is a route using genome-wide association study (GWAS) and genomic selection to modify SnRK/TOR activity ratios and their downstream pathways and products. This has been demonstrated in rice by up-regulating a transcription factor that represses a specific TPP activity, thereby increasing T-6-P and sucrose (Fig. 3). Plants with this up-regulated NAC transcription factor and higher levels of T-6-P (Li *et al.*, 2022) have increased numbers of tillers, larger panicles, more grain, and higher grain yields, similar to the rice plants expressing higher amounts of TOR described above (Bakshi *et al.*, 2017), as predicted. There is also the option to spray T-6-P, or derivatives, at relevant stages of spike and grain development to achieve similar results and gains in grain yield, as has been demonstrated in wheat (Griffiths *et al.*, 2016; Paul *et al.*, 2018a). The discoveries of metabolites that correlate with yields provides opportunities to select lines with the desired levels of metabolites and hence yield potential (Dreccer *et al.*, 2014).

#### Modifying transcription factors downstream of TOR

This review has focused on TOR as an upstream regulator of meristem and spike development because it is a master controller of many, if not all, the principal processes via phosphorylation cascades in spike development. Clearly, altering the regulation of downstream processes, especially via transcription factors that control the induction or repression of large numbers of genes and pathways, also provides attractive candidates for increasing spikelet and fertile floret numbers, as has already been demonstrated by, for example, Wang *et al.* (2017), Li *et al.* (2018), Backhaus *et al.* (2022), and Thirulogachandar *et al.* (2021, Preprint). This approach has much appeal as an alternative or additional approach to the others listed here. Many transcription factors are now known by their transcription profiles during spike development (Wang *et al.*, 2017; Backhaus *et al.*, 2022) and their cellular functions from studies in Arabidopsis and rice and, therefore, when under the control of carefully chosen promoters or edited to have different expression patterns, can be a source of valuable novelty for improving yields.

#### Modifying phosphorylation interactions

The ratios of growth to stress resilience during development are determined by phosphorylation/dephosphorylation of specific protein targets, although there are many such targets. If key proteins expressed in specific tissues/organs could be mutated such that they are no longer subject to phosphorylation/dephosphorylation changes, then growth/resilience outcomes could be modified. TOR could be one such target and specific kinase cascades activated by transcription factors could be others. Transgenes and/or editing could be used to generate plants with such mutations. It would, however, be necessary to determine the targets for such interventions. I noted earlier the findings of Zhang *et al.* (2014) that wheat lines differing in drought tolerance showed many differences in protein phosphorylation, suggesting that trait variation can be altered by changing protein phosphorylation states.

## Concluding comments

The aim of this review was to build on a large literature describing spike development and effects of stress on yield to provide a framework of underlying biochemistry and cell biology suitable for revealing new focused targets and approaches for testing hypotheses and generating changes in seed yield potential.

Spike development and its optimization in/adjustment to genetic backgrounds and agricultural environments involves a very large number of genes and biochemical systems regulated in time and space. However, they are all likely to be subject to the regulatory phosphorylation/dephosphorylation systems described here. Therefore, a more comprehensive genetic and physiological understanding of the roles and regulation of these systems during spike development opens up new and different biochemical targets for genetic intervention and selection. The capacity to make more florets per spike and prevent florets from aborting is clearly present in wheat. Regulation systems, underpinning the management of growth rates of certain organs and sensitivities to stresses in time and space, would appear to be places where new attention should be focused. While the genetics and developmental biology of spikes is complex, there is a need to find single genes of major effect that can be easily managed in breeding programs. That is why focusing on the master regulators at the top of the pathways driving growth is relevant. However, finding genes that function downstream of the master regulators can also open up the means of designing genes with large positive phenotypic effect. They all need to be explored in depth and at scale, using precision targeting and specific assays.

It can be expected that some genetic interventions/selection based on TOR, while having highly advantageous effects on grain yields, will have some negative attributes. These would need to be addressed by selecting modifier variation in genetic backgrounds to find the optimal trait expression, as was done with the dwarfing genes in breeding the lines that enabled the Green Revolution. While many iterations of the proposed experiments described here may be required to find a desired breakthrough in elite germplasm, such novel approaches are necessary because the current rates of yield gain are failing societies.

## References

[CIT0001] Anand A , Trick HN, Gill BS, Muthukrishnan S. 2003. Stable transgene expression and random gene silencing in wheat. Plant Biotechnology Journal 1, 241–251. 17163901 10.1046/j.1467-7652.2003.00023.x

[CIT0002] Backhaus AE , Lister A, Tomkins M, Adamski NM, Simmonds J, Macaulay IC, Morris RJ, Haerty W, Uauy C. 2022. High expression of VRT2 during wheat spikelet initiation increases the number of rudimentary basal spikelets. Plant Physiology. doi: 10.1093/plphys/kiac156 PMC9237664 35377414

[CIT0003] Baena-González E , Hanson J. 2017. Shaping plant development through the SnRK1–TOR metabolic regulators. Current Opinion in Plant Biology 35, 152–157. 28027512 10.1016/j.pbi.2016.12.004

[CIT0004] Bakshi A , Moin M, Kumar MU, Reddy ABM, Ren M, Datla R, Sidiq EA, Kirti PB. 2017. Ectopic expression of Arabidopsis Target of Rapamycin (AtTOR) improves water-use efficiency and yield potential in rice. Scientific Reports 7, 1–16. 28230163 10.1038/srep42835 PMC5322334

[CIT0005] Beauzamy L , Louveaux M, Hamant O, Boudaoud A. 2015. Mechanically, the shoot apical meristem of Arabidopsis behaves like a shell inflated by a pressure of about 1 MPa. Frontiers in Plant Science 6, 1038. 26635855 10.3389/fpls.2015.01038 PMC4659900

[CIT0006] Beveridge CA , Mathesius U, Rose RJ, Gresshoff PM. 2007. Common regulatory themes in meristem development and whole-plant homeostasis. Current Opinion in Plant Biology 10, 44–51. 17157052 10.1016/j.pbi.2006.11.011

[CIT0007] Boussora F , Allam M, Guasmi F, Ferchichi A, Rutten T, Hansson M, Youssef HM, Börner A. 2019. Spike developmental stages and ABA role in spikelet primordia abortion contribute to the final yield in barley (*Hordeum vulgare* L.). Botanical Studies 60, 13. 31292768 10.1186/s40529-019-0261-2 PMC6620232

[CIT0008] Cao P , Fan W, Li P, Hu Y. 2021. Genome-wide profiling of long noncoding RNAs involved in wheat spike development. BMC Genomics 22, 493. 34210256 10.1186/s12864-021-07851-4 PMC8252277

[CIT0009] Chen X , Ding Y, Yang Y, Song C, Wang B, Yang S, Guo Y, Gong Z. 2021. Protein kinases in plant responses to drought, salt, and cold stress. Journal of Integrative Plant Biology 63, 53–78. 33399265 10.1111/jipb.13061

[CIT0010] Coello P , Martínez-Barajas E. 2014. The activity of SnRK1 is increased in *Phaseolus vulgaris* seeds in response to a reduced nutrient supply. Frontiers in Plant Science 5, 196. 24860586 10.3389/fpls.2014.00196 PMC4030202

[CIT0011] Compant S , Clément C, Sessitsch A. 2010. Plant growth-promoting bacteria in the rhizo- and endosphere of plants: their role, colonization, mechanisms involved and prospects for utilization. Soil Biology and Biochemistry 42, 669–678.

[CIT0012] Crepin N. Rolland F. 2019. SnRK1 activation, signaling, and networking for energy homeostasis. Current Opinion in Plant Biology 51, 29–36. 31030062 10.1016/j.pbi.2019.03.006

[CIT0013] Demesa-Arevalo E , Abraham-Juarez MJ, Xu X, Bartlett M, Jackson D. 2021. Maize RAMOSA3 accumulates in nuclear condensates enriched in RNA POLYMERASE II isoforms during the establishment of axillary meristem determinacy. bioRxiv doi: 10.1101/2021.04.06.438639 [Preprint].

[CIT0014] Dobrenel T , Mancera-Martinez E, Forzani C, et al. 2016. The Arabidopsis TOR kinase specifically regulates the expression of nuclear genes coding for plastidic ribosomal proteins and the phosphorylation of the cytosolic ribosomal protein S6. Frontiers in Plant Science 7, 1611. 27877176 10.3389/fpls.2016.01611 PMC5100631

[CIT0015] Dong P , Xiong F, Que Y, Wang K, Yu L, Li Z, Maozhi R. 2015. Expression profiling and functional analysis reveals that TOR is a key player in regulating photosynthesis and phytohormone signaling pathways in Arabidopsis. Frontiers in Plant Science 6, 677. 26442001 10.3389/fpls.2015.00677 PMC4561354

[CIT0016] Draeger T , Moore G. 2017. Short periods of high temperature during meiosis prevent normal meiotic progression and reduce grain number in hexaploid wheat (*Triticum aestivum* L.). Theoretical and Applied Genetics 130, 1785–1800. 28550436 10.1007/s00122-017-2925-1 PMC5565671

[CIT0017] Dreccer MF , Wockner KB, Palta JA, McIntyre CL, Borgognone MG, Bourgault M, Reynolds MJ, Miralles DJ. 2014. More fertile florets and grains per spike can be achieved at higher temperature in wheat lines with high spike biomass and sugar content at booting. Functional Plant Biology 41, 482–495. 32481007 10.1071/FP13232

[CIT0018] Du L , Li S, Ding L, Cheng X, Kang Z, Mao H. 2022. Genome-wide analysis of trehalose-6-phosphate phosphatases (TPP) gene family in wheat indicates their roles in plant development and stress response. BMC Plant Biology 22, 120. 35296251 10.1186/s12870-022-03504-0 PMC8925099

[CIT0019] Figueroa CM , Lunn JE. 2016. A tale of two sugars: trehalose 6-phosphate and sucrose. Plant Physiology 172, 7–27. 27482078 10.1104/pp.16.00417 PMC5074632

[CIT0020] Fischer RA. 2011. Wheat physiology: a review of recent developments. Crop and Pasture Science 62, 95–114.

[CIT0021] Fu L , Liu Y, Qin G, et al. 2021. The TOR–EIN2 axis mediates nuclear signalling to modulate plant growth. Nature 591, 288–292. 33658715 10.1038/s41586-021-03310-y

[CIT0022] Ghiglione HO , Gonzalez FG, Serrago R, Maldonado SB, Chilcott C, Curá JA, Miralles DJ, Zhu T, Casal JJ. 2008. Autophagy regulated by day length determines the number of fertile florets in wheat. The Plant Journal 55, 1010–1024. 18547393 10.1111/j.1365-313X.2008.03570.x

[CIT0023] Gol L , Tomé F, von Korff M. 2017. Floral transitions in wheat and barley: interactions between photoperiod, abiotic stresses, and nutrient status. Journal of Experimental Botany 68, 1399–1410. 28431134 10.1093/jxb/erx055

[CIT0024] González FG , Miralles DJ, Slafer GA. 2011. Wheat floret survival as related to pre-anthesis spike growth. Journal of Experimental Botany 62, 4889–4901. 21705386 10.1093/jxb/err182

[CIT0025] Griffiths CA , Sagar R, Geng Y, et al. 2016. Chemical intervention in plant sugar signalling increases yield and resilience. Nature 540, 574–578. 27974806 10.1038/nature20591

[CIT0026] Guo Z , Chen D, Alqudah A, Röder M, Ganal M, Schnurbusch T. 2016a. Genome-wide association analyses of 54 traits identified multiple loci for the determination of floret fertility in wheat. New Phytologist 214, 257–270. 27918076 10.1111/nph.14342

[CIT0027] Guo Z , Chen D, Röder MS, Ganal MW, Schnurbusch T. 2018a. Genetic dissection of pre-anthesis sub-phase durations during the reproductive spike development of wheat. The Plant Journal 95, 909–918. 10.1111/tpj.13998 29906301

[CIT0028] Guo Z , Chen D, Schnurbusch T. 2018c. Plant and floret growth at distinct developmental stages during the stem elongation phase in wheat. Frontiers in Plant Science 9, 330. 29599792 10.3389/fpls.2018.00330 PMC5863346

[CIT0029] Guo Z , Schnurbusch T. 2015. Variation of floret fertility in hexaploid wheat revealed by tiller removal. Journal of Experimental Botany 66, 5945–5958. 26157170 10.1093/jxb/erv303 PMC4566983

[CIT0030] Guo Z , Slafer GA, Schnurbusch T. 2016b. Genotypic variation in spike fertility traits and ovary size as determinants of floret and grain survival rate in wheat. Journal of Experimental Botany 67, 4221–4230. 27279276 10.1093/jxb/erw200 PMC5301927

[CIT0031] Guo Z , Zhao Y, Röder MS, Reif JC, Ganal MW, Chen D, Schnurbusch T. 2018b. Manipulation and prediction of spike morphology traits for the improvement of grain yield in wheat. Scientific Reports 8, 1–10. 30258057 10.1038/s41598-018-31977-3 PMC6158183

[CIT0032] Henriques R , Bögre L, Horváth B, Magyar Z. 2014. Balancing act: matching growth with environment by the TOR signalling pathway. Journal of Experimental Botany 65, 2691–2701. 24567496 10.1093/jxb/eru049

[CIT0033] Huang H , Ullah F, Zhou DX, Yi M, Zhao Y. 2019. Mechanisms of ROS regulation of plant development and stress responses. Frontiers in Plant Science 10, 800. 31293607 10.3389/fpls.2019.00800 PMC6603150

[CIT0034] Islam M , Inoue T, Hiraide M, et al. 2021. Activation of SnRK2 by Raf-like kinase ARK represents a primary mechanism of ABA and abiotic stress responses. Plant Physiology 185, 533–546. 33655297 10.1093/plphys/kiaa046 PMC8133623

[CIT0035] Janocha D , Pfeiffer A, Dong Y, Novák O, Strnad M, Ryabova LA, Lohmann JU. 2022. TOR kinase controls shoot development by translational regulation of cytokinin catabolic enzymes. bioRxiv doi: 10.1101/2021.07.29.454319 [Preprint].

[CIT0036] Kamiyama Y , Hirotani M, Ishikawa S, et al. 2021a. Arabidopsis group C Raf-like protein kinases negatively regulate abscisic acid signaling and are direct substrates of SnRK2. Proceedings of the National Academy of Sciences, USA 118, e2100073118. 10.1073/pnas.2100073118 PMC8325330 34282011

[CIT0037] Kamiyama Y , Katagiri S, Umezawa T. 2021b. Growth promotion or osmotic stress response: how SNF1-related protein kinase 2 (SnRK2) kinases are activated and manage intracellular signaling in plants. Plants 10, 1443. 34371646 10.3390/plants10071443 PMC8309267

[CIT0038] Kazan K , Lyons R. 2016. The link between flowering time and stress tolerance. Journal of Experimental Botany 67, 47–60. 26428061 10.1093/jxb/erv441

[CIT0039] Kim KM , Kim KH, Cheong YK, et al. 2019. ‘Taejoong’ a wheat variety with good noodle quality, red grain wheat, long spike, and moderate resistance to fusarium head blight. Korean Society of Breeding Science 51, 454–461.

[CIT0040] Kirby EJM. 1988. Analysis of leaf, stem and ear growth in wheat from terminal spikelet stage to anthesis. Field Crops Research 18, 127–140.

[CIT0041] Kulma A , Villadsen D, Campbell DG, Meek SE, Harthill J, Nielsen TH, MacKintosh C. 2004. Phosphorylation and 14-3-3 binding of Arabidopsis 6-phosphofructo-2-kinase/fructose-2,6-bisphosphatase. The Plant Journal 37, 654–667. 14871307 10.1111/j.1365-313x.2003.01992.x

[CIT0042] Lawlor DW , Paul MJ. 2014. Source/sink interactions underpin crop yield: the case for trehalose 6-phosphate/SnRK1 in improvement of wheat. Frontiers in Plant Science 5, 418. 25202319 10.3389/fpls.2014.00418 PMC4142875

[CIT0043] Li T , Deng G, Tang Y, et al. 2021. Identification and validation of a novel locus controlling spikelet number in bread wheat (*Triticum aestivum* L.). Frontiers in Plant Science 12, 611106. 33719283 10.3389/fpls.2021.611106 PMC7952655

[CIT0044] Li X , Cai W, Liu Y, Li H, Fu L, Liu Z, Xu L, Liu H, Xu T, Xiong Y. 2017. Differential TOR activation and cell proliferation in Arabidopsis root and shoot apexes. Proceedings of the National Academy of Sciences, USA 114, 2765–2770. 10.1073/pnas.1618782114 PMC5347562 28223530

[CIT0045] Li Y , Fu X, Zhao M, Zhang W, Li BO, An D, Li J, Zhang A, Liu R, Liu X. 2018. A genome-wide view of transcriptome dynamics during early spike development in bread wheat. Scientific Reports 8, 15338. 30337587 10.1038/s41598-018-33718-y PMC6194122

[CIT0046] Li Z , Wei X, Tong X, et al. 2022. The OsNAC23–Tre6P–SnRK1a feed-forward loop regulates sugar homeostasis and grain yield in rice. Molecular Plant 15, 706–722. 35093592 10.1016/j.molp.2022.01.016

[CIT0047] Lichthardt C , Chen TW, Stahl A, Stützel H. 2020. Co-evolution of sink and source in the recent breeding history of winter wheat in Germany. Frontiers in Plant Science 10, 1771. 32117340 10.3389/fpls.2019.01771 PMC7019858

[CIT0048] Lin L , Wu J, Jiang M, Wang Y. 2021. Plant mitogen-activated protein kinase cascades in environmental stresses. International Journal of Molecular Sciences 22, 1543. 33546499 10.3390/ijms22041543 PMC7913722

[CIT0049] Liu J , Xu Z, Fan X, Zhou Q, Cao J, Wang F, Ji G, Yang L, Feng D, Wang T. 2018. A genome-wide association study of wheat spike related traits in China. Frontiers in Plant Science 9, 1584. 30429867 10.3389/fpls.2018.01584 PMC6220075

[CIT0050] Lozano-Juste J , Alrefaei AF, Rodriguez PL. 2020 Plant osmotic stress signaling: MAPKKKs meet SnRK2s. Trends in Plant Science 25, 1179–1182. 32972846 10.1016/j.tplants.2020.09.003

[CIT0051] Lyra DH , Griffiths CA, Watson A, Joynson R, Molero G, Igna AA, Hassani P, Reynolds MP, Hall A, Paul MJ. 2021. Gene-based mapping of trehalose biosynthetic pathway genes reveals association with source- and sink-related yield traits in a spring wheat panel. Food and Energy Security 10, e292. 34594548 10.1002/fes3.292 PMC8459250

[CIT0052] Ma C , Zhou J, Chen G, Bian Y, Lv D, Li X, Wang Z, Yan Y. 2014. iTRAQ-based quantitative proteome and phosphoprotein characterization reveals the central metabolism changes involved in wheat grain development. BMC Genomics 15, 1–20. 25427527 10.1186/1471-2164-15-1029 PMC4301063

[CIT0053] Ma Y , Cao J, He J, Chen Q, Li X, Yang Y. 2018. Molecular mechanism for the regulation of ABA homeostasis during plant development and stress responses. International Journal of Molecular Sciences 19, 3643. 30463231 10.3390/ijms19113643 PMC6274696

[CIT0054] Mao X , Li Y, Rehman SU, Miao L, Zhang Y, Chen X, Yu C, Wang J, Li C, Jing R. 2020. The sucrose non-fermenting 1-related protein kinase 2 (SnRK2) genes are multifaceted players in plant growth, development and response to environmental stimuli. Plant and Cell Physiology 61, 225–242. 31834400 10.1093/pcp/pcz230

[CIT0055] Mao X , Zhang H, Tian S, Chang X, Jing R. 2010. TaSnRK2.4, an SNF1-type serine/threonine protein kinase of wheat (*Triticum aestivum* L.), confers enhanced multistress tolerance in Arabidopsis. Journal of Experimental Botany 61, 683–696. 20022921 10.1093/jxb/erp331 PMC2814103

[CIT0056] Mao Y , Botella R, Liu Y, Zhu JK. 2019. Gene editing in plants: progress and challenges. National Science Review 6, 421–437. 34691892 10.1093/nsr/nwz005 PMC8291443

[CIT0057] Margalha L , Confraria A, Baena-González E. 2019. SnRK1 and TOR: modulating growth–defense trade-offs in plant stress responses. Journal of Experimental Botany 70, 2261–2274. 30793201 10.1093/jxb/erz066

[CIT0058] Martínez-Barajas E , Delatte T, Schluepmann H, de Jong GJ, Somsen GW, Nunes C, Primavesi LF Coello C, Mitchell RAC, Paul MJ. 2011. Wheat grain development is characterized by remarkable trehalose 6-phosphate accumulation pregrain filling: tissue distribution and relationship to SNF1-related protein kinase1 activity. Plant Physiology 156, 373–381. 21402798 10.1104/pp.111.174524 PMC3091070

[CIT0059] Mishra S , Sharma P, Singh R, Tiwari R. Singh GP. 2021. Genome-wide identification and expression analysis of sucrose nonfermenting-1-related protein kinase (SnRK) genes in *Triticum aestivum* in response to abiotic stress. Scientific Reports 11, 1–17. 34795369 10.1038/s41598-021-99639-5 PMC8602265

[CIT0060] Mondal S , Dutta S, Crespo-Herrera L, Huerta-Espino J, Braun HJ, Singh RP. 2020. Fifty years of semi-dwarf spring wheat breeding at CIMMYT: grain yield progress in optimum, drought and heat stress environments. Field Crops Research 250, 107757.

[CIT0061] Murchie EH , Reynolds R, Slafer GA, et al. 2022. A ‘wiring diagram’ for source strength traits impacting wheat yield potential. Journal of Experimental Botany. doi: 10.1093/jxb/erac415 PMC9786870 36264277

[CIT0062] Nakashima K , Fujita Y, Kanamori N, et al. 2009. Three Arabidopsis SnRK2 protein kinases, SRK2D/SnRK2.2, SRK2E/SnRK2.6/OST1 and SRK2I/SnRK2.3, involved in ABA signaling are essential for the control of seed development and dormancy. Plant and Cell Physiology 50, 1345–1363. 19541597 10.1093/pcp/pcp083

[CIT0063] Nuccio ML , Wu J, Mowers R, et al. 2015. Expression of trehalose-6-phosphate phosphatase in maize ears improves yield in well-watered and drought conditions. Nature Biotechnology 33, 862–869. 10.1038/nbt.3277 26473199

[CIT0064] Nukarinen E , Nägele T, Pedrotti L, et al. 2016. Quantitative phosphoproteomics reveals the role of the AMPK plant ortholog SnRK1 as a metabolic master regulator under energy deprivation. Scientific Reports 6, 31697. 27545962 10.1038/srep31697 PMC4992866

[CIT0065] Nunes C , Primavesi LF, Patel MK, Martinez-Barajas E, Powers SJ, Sagar R, Fevereiro PS, Davis BG, Paul MJ. 2013. Inhibition of SnRK1 by metabolites: tissue-dependent effects and cooperative inhibition by glucose 1-phosphate in combination with trehalose 6-phosphate. Plant Physiology and Biochemistry 63, 89–98. 23257075 10.1016/j.plaphy.2012.11.011

[CIT0066] Paul MJ , Gonzalez-Uriarte A, Griffiths CA, Hassani-Pak K. 2018a. The role of trehalose 6-phosphate in crop yield and resilience. Plant Physiology 177, 12–23. 29592862 10.1104/pp.17.01634 PMC5933140

[CIT0067] Paul MJ , Nuccio ML, Basu SS. 2018b. Are GM crops for yield and resilience possible? Trends in Plant Science 23, 10–16. 28969999 10.1016/j.tplants.2017.09.007

[CIT0068] Paul MJ , Watson A, Griffiths CA. 2020. Trehalose 6-phosphate signalling and impact on crop yield. Biochemical Society Transactions 48, 2127–2137. 33005918 10.1042/BST20200286 PMC7609034

[CIT0069] Pizzio GA , Rodriguez PL. 2022. Dual regulation of SnRK2 signaling by Raf-like MAPKKKs. Molecular Plant 15, 1260–1262. 35810328 10.1016/j.molp.2022.07.002

[CIT0070] Pretini N , Alonso MP, Vanzetti LS, Pontaroli AC, González FG. 2021. The physiology and genetics behind fruiting efficiency: a promising spike trait to improve wheat yield potential. Journal of Experimental Botany 72, 3987–4004. 33681978 10.1093/jxb/erab080

[CIT0071] Ray DK , Mueller ND, West PC, Foley JA. 2013. Yield trends are insufficient to double global crop production by 2050. PLoS One 8, e66428. 23840465 10.1371/journal.pone.0066428 PMC3686737

[CIT0072] Reynolds MP , Slafer GA, Foulkes JM, et al. 2022. A wiring diagram to integrate physiological traits of wheat yield potential. Nature Food 3, 318–324. 10.1038/s43016-022-00512-z 37117579

[CIT0073] Rimbert H , Darrier B, Navarro J, Kitt J, Choulet F, Leveugle M, Duarte T, Riviere N, Eversole K. 2018. High throughput SNP discovery and genotyping in hexaploid wheat. PLoS One 13, e0186329. 29293495 10.1371/journal.pone.0186329 PMC5749704

[CIT0074] Rosenberger CL , Chen J. 2018. To grow or not to grow: TOR and SnRK2 coordinate growth and stress response in Arabidopsis. Molecular Cell 69, 3–4. 29304332 10.1016/j.molcel.2017.12.013

[CIT0075] Sakuma S , Golan G, Guo Z, et al. 2019. Unleashing floret fertility in wheat through the mutation of a homeobox gene. Proceedings of the National Academy of Sciences, USA 116, 5182–5187. 10.1073/pnas.1815465116 PMC6421441 30792353

[CIT0076] Sakuma S , Schnurbusch T. 2020. Of floral fortune: tinkering with the grain yield potential of cereal crops. New Phytologist 225, 1873–1882. 31509613 10.1111/nph.16189

[CIT0077] Schepetilnikov M , Ryabova LA. 2018. Recent discoveries on the role of TOR (Target of Rapamycin) signaling in translation in plants. Plant Physiology 176, 1095–1105. 29122989 10.1104/pp.17.01243 PMC5813564

[CIT0078] Schulze WX. 2010. Proteomics approaches to understand protein phosphorylation in pathway modulation. Current Opinion in Plant Biology 13, 279–286. 10.1016/j.pbi.2009.12.008 20097120

[CIT0079] Sesma A , Castresana C, Castellano MM. 2017. Regulation of translation by TOR, eIF4E and eIF2α in plants: current knowledge, challenges and future perspectives. Frontiers in Plant Science 8, 644. 28491073 10.3389/fpls.2017.00644 PMC5405063

[CIT0080] Shewry PR , Evers AD, Bechtel DB, Abecassis J. 2009. Development, structure, and mechanical properties of the wheat grain. In: Khan K, Shewry P, eds. Wheat: chemistry amd technology, 4th edn. St Paul, MN: AACC, 51–96.

[CIT0081] Shewry PR , Mitchell RA, Tosi P, Wan Y, Underwood C, Lovegrove A, Freeman J, Toole GA, Mills EC, Ward JL. 2012. An integrated study of grain development of wheat (cv. Hereward). Journal of Cereal Science 56, 21–30.

[CIT0082] Shi L , Wu Y, Sheen J. 2018. TOR signaling in plants: conservation and innovation. Development 145, dev160887. 29986898 10.1242/dev.160887 PMC6053665

[CIT0083] Shin DH , Choi MG, Kang CS, Park CS, Choi SB, Park YI. 2015. Preferential expression of cell elongation-related genes in leaves of the new elite wheat line Iksan370 with large spikes. Plant Biotechnology Reports 9, 97–105.

[CIT0084] Slafer GA , Foulkes MJ, Reynolds M, Murchie EH, Carmo-Silva E, Flavell R, Gwyn J, Sawkins M, Griffiths S. 2022. A ‘wiring diagram’ for sink strength traits impacting wheat yield potential. Journal of Experimental Botany. doi: 10.1093/jexb/erac410 PMC9786893 36334052

[CIT0085] Sugden C , Donaghy PG, Halford NG, Hardie DG. 1999. Two SNF1-related protein kinases from spinach leaf phosphorylate and inactivate 3-hydroxy-3-methylglutaryl-coenzyme A reductase, nitrate reductase, and sucrose phosphate synthase in vitro. Plant Physiology 120, 257–274. 10318703 10.1104/pp.120.1.257 PMC59258

[CIT0086] Sun Z , Feng Z, Ding Y, et al. 2022. RAF22, ABI1 and OST1 form a dynamic interactive network that optimizes plant growth and responses to drought stress in Arabidopsis. Molecular Plant 15, 1192–1210. 35668674 10.1016/j.molp.2022.06.001

[CIT0087] Thiel J , Koppolu R, Trautewig C, et al. 2021. Transcriptional landscapes of floral meristems in barley. Science Advances 7, eabf0832. 33910893 10.1126/sciadv.abf0832 PMC8081368

[CIT0088] Thirulogachandar V , Govind G, Hensel G, et al. 2021. Dosage of duplicated and antifunctionalized homeobox proteins influences spikelet development in barley. bioRxiv doi: 10.1101/2021.11.08.467769 [Preprint].

[CIT0089] Tuan PA , Yamasaki Y, Kanno Y, Seo M, Ayele BT. 2019. Transcriptomics of cytokinin and auxin metabolism and signaling genes during seed maturation in dormant and non-dormant wheat genotypes. Scientific Reports 9, 3983. 30850728 10.1038/s41598-019-40657-9 PMC6408541

[CIT0090] Ur Rehman S , Wang J, Chang X, Zhang X, Mao X, Jing R. 2019. A wheat protein kinase gene TaSnRK2.9-5A associated with yield contributing traits. Theoretical and Applied Genetics 132, 907–919. 30519711 10.1007/s00122-018-3247-7 PMC6449320

[CIT0091] Van Leene J , Han C, Gadeyne A, et al. 2019. Capturing the phosphorylation and protein interaction landscape of the plant TOR kinase. Nature Plants 5, 316–327. 30833711 10.1038/s41477-019-0378-z

[CIT0092] Volpato L , Pinto F, González-Pérez L, Thompson IG, Borém A, Reynolds M, Gerard B, Molero G, Rodrigues FA Jr. 2021. High throughput field phenotyping for plant height using UAV-based RGB imagery in wheat breeding lines: feasibility and validation. Frontiers in Plant Science 12, 185. 10.3389/fpls.2021.591587 PMC7921806 33664755

[CIT0093] Vos R , Bellù LG. 2019. Global trends and challenges to food and agriculture into the 21st century. In: Campanhola C, Pandey S, eds. Sustainable food and agriculture: an integated approach. FAO/Elsevier, 11–30

[CIT0094] Wang P , Zhao Y, Li Z, et al. 2018. Reciprocal regulation of the TOR kinase and ABA receptor balances plant growth and stress response. Molecular Cell 69, 100–112. 29290610 10.1016/j.molcel.2017.12.002 PMC5772982

[CIT0095] Wang X , Bian Y, Cheng K, Gu LF, Ye M, Zou H, Sun SS, He JX. 2013. A large-scale protein phosphorylation analysis reveals novel phosphorylation motifs and phosphoregulatory networks in Arabidopsis. Journal of Proteomics 78, 486–498. 23111157 10.1016/j.jprot.2012.10.018

[CIT0096] Wang Y , Yu H, Tian C, Sajjad M, Gao C, Tong Y, Wang X, Jiao Y. 2017. Transcriptome association identifies regulators of wheat spike architecture. Plant Physiology 175, 746–757. 28807930 10.1104/pp.17.00694 PMC5619896

[CIT0097] Watson A , Ghosh S, Williams MJ, et al. 2018. Speed breeding is a powerful tool to accelerate crop research and breeding. Nature Plants 4, 23–29. 29292376 10.1038/s41477-017-0083-8

[CIT0098] Whingwiri EE , Stern WR. 1982. Floret survival in wheat: significance of the time of floret initiation relative to terminal spikelet formation. Journal of Agricultural Science 98, 257–268.

[CIT0099] Wu Y , Shi L, Li L, Fu L, Liu Y, Xiong Y, Sheen J. 2019. Integration of nutrient, energy, light, and hormone signalling via TOR in plants. Journal of Experimental Botany 70, 2227–2238. 30715492 10.1093/jxb/erz028 PMC6463029

[CIT0100] Zhang H , Li W, Mao X, Jing R, Jia H. 2016. Differential activation of the wheat SnRK2 family by abiotic stresses. Frontiers in Plant Science 7, 420. 27066054 10.3389/fpls.2016.00420 PMC4814551

[CIT0101] Zhang M , Lv D, Ge P, Bian Y, Chen G, Zhu G, Yan Y. 2014. Phosphoproteome analysis reveals new drought response and defense mechanisms of seedling leaves in bread wheat (*Triticum aestivum* L.). Journal of Proteomics 109, 290–308. 25065648 10.1016/j.jprot.2014.07.010

[CIT0102] Zhang P , He Z, Tian X, et al. 2017. Cloning of TaTPP-6AL1 associated with grain weight in bread wheat and development of functional marker. Molecular Breeding 37, 78.

[CIT0103] Zhang W , Wang J, Huang Z, Mi L, Xu K, Wu J, Fan Y, Ma S, Jiang D. 2019. Effects of low temperature at booting stage on sucrose metabolism and endogenous hormone contents in winter wheat spikelet. Frontiers in Plant Science 10, 498. 31057594 10.3389/fpls.2019.00498 PMC6482243

[CIT0104] Zhang Z , Zhu JY, Roh J, Marchive C, Kim SK, Meyer C, Sun Y, Wang W, Wang ZY. 2016. TOR signaling promotes accumulation of BZR1 to balance growth with carbon availability in Arabidopsis. Current Biology 26, 1854–1860. 27345161 10.1016/j.cub.2016.05.005 PMC5126233

